# Factors associated with tungiasis among school-age children in Kwale County, rural Kenya

**DOI:** 10.1093/inthealth/ihac013

**Published:** 2022-04-07

**Authors:** Judy Mwai, Diana Nyole, Mohamed H Abdi, Jarim Omogi

**Affiliations:** Kenya Medical Research Institute-CPHR; ITROMID-Jomo Kenyatta University of Agriculture and Technology; Kenya Medical Research Institute-CPHR; Amref International University

**Keywords:** jigger, prevention and control, school-age children, tungiasis

## Abstract

**Background:**

The parasitic disease tungiasis, caused by the flea *Tunga penetrans*, remains an important public health problem among children and the elderly. The study assessed the factors influencing prevention and control of tungiasis infection among school-age children in Kwale County, Kenya.

**Methods:**

A cross-sectional survey was conducted in five villages in Lunga Lunga subcounty among 538 children ages 5–14 y. The study employed a mobile application tool in collecting sociodemographic, knowledge, perception and practice data on prevention and control of tungiasis with frequencies and bivariate and multivariate regression analysis used.

**Results:**

The prevalence of tungiasis was found to be 62.1% (328/528), with fathers’ education level, place of residence and wearing shoes being factors associated with infection. Those who wore shoes were less likely to be infected compared with those who did not (odds ratio 0.059 [95% confidence interval 0.29 to 0.12]). Children living in Dzombo B and Kinyungu were less likely to be infected with tungiasis compared with those living in Bandu, holding other factors constant.

**Conclusion:**

Creating awareness of the cause of tungiasis remains of key public importance. Hygiene promotion, including wearing of shoes and the general cleanliness of the environment at the community level, needs to be implemented.

## Introduction

Tungiasis is an ectoparasitosis that is caused by infection with *Tunga penetrans*, also known as jigger flea or sand flea.^1^ The highly neglected parasitic skin disease is known to inflict pain and suffering on millions of impoverished people in South America, the Caribbean and sub-Saharan Africa (SSA). Research on tungiasis is scant and most of the publications originate from South America, with literature showing that very little work has been done in SSA.^2^ According to the World Health Organization (WHO), >20 million individuals are estimated to be at risk in the WHO Region of the Americas alone.^3^ In endemic areas, 95–98% of all tungiasis lesions occur on the feet.^4^

The literature shows that school-age children who were diagnosed with parasitic worm infections had lowered school attendance and cognitive and physical development, which was found to improve after treatment.^5^ It has also been shown to reduce the learning ability of children, retard their growth and reduce their potential for future employability.^5^

Besides socio-economic factors, male gender, age <15 y and older age groups, a sandy floor inside the house, no regular use of footwear and the presence of animals on the compound are associated with a higher prevalence and more severe morbidity.^6^

Tungiasis thrives where living conditions are precarious, such as villages located near remote beaches, communities in the rural hinterland and shanty towns of big cities.^3^ In these settings, the poorest of the poor carry the highest burden of the disease.^3^ In resource-poor urban neighbourhoods and rural communities, prevalence may be as high as 60% in the general population and up to 80% in children. Elderly people and children ages 5–14 y, particularly boys, are at highest risk.^3^ People with disabilities are also highly vulnerable to infection.^3^ Overall, the disease is usually not perceived as an important condition, not by the populations affected or by decision makers and health personnel, and there are often no district or countrywide control programs.^6^

According to pilot investigations, tungiasis is highly prevalent in parts of the Coast, Nyanza, Rift Valley, Western and Central regions of Kenya. However, sustainable control measures against tungiasis can only be developed if the epidemiological situation is well understood.^7^ Unhygienic conditions have been identified as the major cause of tungiasis in Kenya.^8^ In addition, soil factors such as organic matter content, moisture, pH, colour and texture have been suggested to influence the prevalence of tungiasis by up to 33%.^5^ Soil factors were also found to influence the *T. penetrans* population by up to 39.7%.^5,9^ Although such information is already available, more work on epidemiology, especially in vulnerable age groups such as children and the aged, in other endemic regions in Kenya need to be pursued.

The study was conducted in Lunga Lunga subcounty, which is located in extreme southeastern Kenya, at the border with the Republic of Tanzania. The location was chosen given the high poverty level in the subcounty according to the Kwale County government^10^ In addition, the location was chosen due to its proximity to wildlife and the fact that the community keeps domesticated animals. It is suspected that the ecological determinants of tungiasis include a complex web of factors including humans, domesticated animals and wildlife.^11^ A major prerequisite to obtain community support for control measures is to understand the knowledge and practices of the individuals affected. Studies on several parasitic diseases, such as lymphatic filariasis and malaria, have shown that ignorance and incorrect concepts about transmission, disease sequelae and treatment options may lead to negligence in prevention, reluctance in accepting treatment and failure to support control measures.^12^ The study was therefore designed to determine the factors that influence and control tungiasis infection among school-age children in Lunga Lunga subcounty of Kwale County, Kenya. This study is important given the practical sustainable solutions it proposes that will not only be of assistance to the county government of Kwale, but also policymakers at the national level.

## Methods

### Study area

The study was conducted in Lunga Lunga subcounty of Kwale County, Kenya in five locations: Bandu, Dzombo A, Dzombo B, Kinyungu and Menza Mwenye, which is largely a rural area. Lunga Lunga has a population of 198 423 and an area of 2803.80 km^2^. The subcounty has a total of four wards: Pongwe, Dzombo, Mwereni and Vanga. The subcounty has a total of 24 health facilities, among them Lunga Lunga Subcounty Hospital, which is a level 4 hospital. The main socio-economic activities in the area include agriculture, fishing and tourism. Tungiasis is endemic in coastal counties of Kenya, affecting mostly school-age children and the elderly.^4,11^

### Study design and population

A cross-sectional study was conducted during the high transmission season (dry season 2020). Using a proportion of 0.23, as per the 2019 census,^13^ the population of children ages 5–14 y in Lunga Lunga was assumed to be 84 699. The minimum sample size was determined using the Fisher formula,^14^ where n is the minimum required sample size, z is the standard normal deviation for a 95% confidence interval, p is the estimated prevalence of tungiasis, α is the level of significance and δ is the degree of precision. The prevalence was estimated at 50% for z=1.96, p=0.5, d=0.05, δ=0.05, α=0.05 response rate=95% and design effect=1.5, thus n was estimated 538.

The study targeted children ages 5–14 y who were recruited from their homes following the parents’ consent for the child to participate in the study. Households with no children or with children <5 y or >14 y were excluded.

### Sampling technique

The proportional stratified random sampling (PSRS) method was used to determine the proportion of households’ sampled in each village and to select households with children in primary schools in each village. The PSRS was first used to determine the population in each village that represented a stratum. The total population of the school-age children in the village was divided by the proportion of the population in each village. The method was used to maximize the homogeneity within the villages.

A list of all households with school-age children was obtained from a village register with the help of community health workers and local administrations. The households were then randomly selected from a representative sample of the study. In each household only one child age 5–14 y was selected. Birth order was used in households with more than one child within the selected age group and the oldest child was selected (Table 1).

**Table 1.** Sample size per village

Site no.	Village	Subvillage	Population	Sample, n
1	Bandu	Mirima Maraba	11 021	42
2		Kasemeni	5731	22
3		Pangani	4080	15
4		Bumbani	11 400	43
5	Dzombo A	Perani	7580	29
6		Sega	8400	32
7		Manda	7360	28
8		Mwena	5430	21
9	Dzombo B	Bidinimole	11 900	45
10		Kilimangodo	9800	37
11		Jego	1670	6
12	Kinyungu	Kiwegu	8900	35
13		Vanga	5888	23
14		Majoreni	13 680	54
15	Menza Mwenye	Mzizima	11 548	43
16		Shimoni	14 852	56
17		Wasini/Mkwiro	4612	17
Total			143 852	538

### Data collection

In the selected households, consent was sought from parents/guardians and assent from the pupils to participate in the study. A questionnaire was administered in English or Swahili through a face-to-face interview with the selected household member. The collection and storage of the data was done using tablets and smartphones using the electronic data collection method Open Data Kit (https://opendatakit.org/), which allowed for submission of the data to a secure server.

The questionnaire covered four areas: sociodemographic factors (sex, age, education); housing factors (type of construction of the house, type of flooring material used in the house, sanitary conditions, waste disposal); presence of domestic animals; and knowledge, perceptions and practices related to tungiasis (knowledge on transmission, regular use of footwear, preventive measures, treatment). Pretesting was done in 36 households that were not part of the sampled households but were within the selected villages.

### Statistical analysis

Statistical analysis was done after data validation using Stata version 15 (StataCorp, College Station, TX, USA). Descriptive statistics including mean, standard deviation and frequencies were generated. Tables and bar graphs were used to present the frequencies. Additionally, χ^2^ tests were used to test associations between tungiasis infection and other variables, including demographic characteristics, socio-economic characteristics, presence of domestic animals and knowledge on transmission. Cross-tabulation and was used to describe data and to explain the relationship between tungiasis infection and the above independent variables. Multivariate regression was used to investigate how tungiasis infection was related to knowledge, gender, village and education level, among other independent variables.

## Results

### Sociodemographic characteristics

A total of 538 respondents were interviewed, with the majority in Menza Mwenye (116 [21.6%]), Bandu (115 [21.4%]) and Kinyungu (113 [21%]). The mean age of the children was 10±2.51. More than half (292 [57.2%]) of the respondents’ mothers had a primary level of education, while 111 (20.6%) had not attended school. The fathers’ education level was primary (263 [51.2%]) and secondary (128 [24.9%]). The majority of the respondents’ fathers were farmers (215 [40%]) and business/self-employed (104 [19.3%]) as shown in Table 2.

**Table 2.** Results of cross-tabulation of sociodemographic characteristics and tungiasis infection

Variables	Tungiasis infection			Statistical significance
	Yes, n (%)	No, n (%)	All, n (%)	
Village				
Bandu	91 (79.1)	24 (20.9)	115 (21.4)	χ^2^=155.4, p=0.001
Dzombo A	78 (73/6)	28 (26.4)	106 (19.7)	
Dzombo B	41 (46.6)	47 (53.4)	88 (16.4)	
Kinyungu	18 (15.9)	95 (84.1)	113 (21)	
Menza Mwenye	100 (86.2)	16 (13.8)	116 (21.6)	
Mothers’ education level				0.114 (Fisher's exact test)
None	76 (68.5)	35 (31.5)	111 (24.4)	
Primary	200 (68.5)	92 (31.5)	292 (64.3)	
Secondary	31 (64.6)	17 (35.4)	48 (9.5)	
Tertiary	0 (0)	3 (100)	3 (0.6)	
Fathers’ education level				χ^2^=9.64, p=0.022
None	21 (48.8)	22 (51.1)	43 (9.4)	
Primary	186 (70.7)	77 (29.3)	263 (57.7)	
Secondary	83 (64.8)	45 (35.2)	128 (28.1)	
Tertiary	12 (54.6)	10 (45.4)	22 (4.8)	
Fathers’ occupation				χ^2^=5.17, p=0.270
Employed	64 (70.3)	27 (29.7)	91 (16.9)	
Farmer	131 (60.9)	84 (39.1)	215 (40)	
Business/self employed	59 (56.7)	45 (43.3)	104 (19.3)	
Casual worker	55 (67.9)	26 (32.1)	81 (15.1)	

The major type of house flooring was earth/sand, while type of walls was brick (69.9%) and wood/planks (24.5%). In total, 328 (61%) of the respondents confirmed that a member of their household or themselves had been affected by tungiasis in the previous month. This was regarded as the prevalence of tungiasis since it represented the proportion of school-age children having been affected by tungiasis during the research period.

### Knowledge on tungiasis occurrence

According to 452 (85.9%) and 448 (85.2%) of the respondents, poor hygiene and dust, respectively, are the causes of tungiasis, with only 1 (0.2%) mentioning correctly that it is caused by fleas while 74 (13.7%) correctly mentioned that tungiasis breeds under the skin. Others mentioned dusty soils (58.2%), trees (25%), water (1.3%) and flowers (1.1%). Only 29 (6.9%) of the respondents were aware that tungiasis can be transmitted through domestic animals, while 235 (56.2%) identified infected persons correctly. The knowledge on the symptoms of tungiasis were correctly identified and included itching 421 (81.7%), pain 409 (79.4%) and swelling 391 (75.9%). Others included blackspot (306 [59.4%]) and watery discharge (291 [56.5%]). Others mentioned vomiting (2 [0.4%]) and severe headache (8 [1.6%]). On how to prevent infection with tungiasis, the respondents correctly identified good hygiene (448 [84.2%]) and wearing shoes (326 [61.3%]) as some of the practices. Others mentioned avoiding endemic areas (115 [21.6%]), daily inspection of the feet (75 [14.1%]) and plastering homes (7 [1.3%]).

Using Bloom cut-off points of 80–100% for high knowledge, positive attitude and good practice, 60–79% for moderate knowledge and ≤59 for poor knowledge, only 22.1% and 13.5% had high and moderate knowledge, respectively, while 64.4% had poor knowledge. No association was found between knowledge and tungiasis infection.

### Perception towards tungiasis

A total of 344 (63.9%) respondents indicated that tungiasis is a serious problem in their village due to poor personal hygiene (251 [79.4%]), inadequate household spraying (143 [45.2%]) and not wearing closed shoes (139 [44%]).

According to the respondents, tungiasis leads to a lack of sleep (87.1%), absence from school (56.9%) and stigmatization (42.6%). Others opined that it has made them not play with other children (39.5%), unable to concentrate in class (38.6%) and unable to do household chores (30.6%).

A total of 334 (37.1%) respondents indicated that tungiasis affected their relationships with their friends or relatives, as 58.6% indicated that their friends refuse to play with them, their friends perceived them as dirty (30.4%), their friends avoid coming close to them (29.9%) and their friends avoid talking to them (7.2%).

Overall, the respondents indicated that they are perceived as dirty (91.1%), poor (27.5%), bewitched (6.3%) and lazy (2.1%) by their neighbours since they were infected with tungiasis.

### Individual practices towards disease occurrence

When infected with tungiasis, the major step the children took was extraction/removal by themselves (75.2%) and telling a parent or guardian (68%). A total of 409 (76%) households were found to be keeping domestic animals, with goats (66%), poultry (64.3%) and cattle (46.7%) being the top three animals kept. When asked what they do whenever they are infected with tungiasis, 381 (71.1%) said that they both remove and apply products, 254 (47.4%) only remove, while 159 (30%) apply products without removing. Some of the products applied included kerosene, used engine oil and household insecticides.

Less than one-third (22%) of the respondents wore shoes consistently, with 72.6% wearing shoes only occasionally. A strong association was found between wearing shoes and tungiasis infection, with the odds of being infected among those who do not wear shoes being 1.9 times higher compared with those who do wear shoes.

Further analysis revealed a significance between village and tungiasis infection and between wearing shoes and tungiasis infection, shown in table 2. Further analysis using multivariate analysis showed that children living in Dzombo A, Dzombo B and Kinyungu were less likely to be infected with tungiasis compared with those living in Bandu, holding other factors constant, as shown in table 3.

**Table 3.** Results of logistic regression in the final model showing significant effects on tungiasis infection (final model included only significant variables at p=0.05)

Variables	B	SE	p>|z|	Exp(B)	95% confidence interval
0 (base outcome)					
1					
Village					
Dzombo A	0.81	0.35	0.020	0.45	0.31 to 1.31
Dzombo B	3.50	0.37	0.000	0.03	0.09 to 0.37
Kinyungu	1.96	0.34	0.000	0.14	0.02 to 0.11
Menza Mwenye	0.50	0.35	0.158	0.61	0.58 to 2.17
Bandu					
Fathers’ education					
Primary	1.96	0.75	0.07		0.93 to 4.17
Secondary	2.04	0.87	0.09		0.89 to 4.71
Tertiary	0.79	0.45	0.69		0.26 to 2.43
None*					
Wearing shoes					
Yes	2.83	0.361	0.00	0.059	0.029 to 0.120
No					

## Discussion

Tungiasis is a parasitic infection whose transmission is associated with poor or inadequate sanitation. The objective of this study was to determine the prevalence and factors associated with prevention and control of tungiasis among school-age children in coastal rural Kenya. This study showed a high prevalence (61%) of tungiasis in Lunga Lunga subcounty, Kwale County, Kenya. Tungiasis infection was associated with the fathers’ education level, residence and not wearing shoes.

Prevalence of Tungiasis in Kwale County was approximately 61%, similar to a study conducted in in children in Ethiopia.^15^ The high prevalence could be a result of the children spending most of their time playing on the ground, as suggested by Wiese and others.^4^ The high prevalence could also be a result of the floor of the home, as more than two-thirds were earth/sand. In contrast, a population-based study in Nigeria had a slightly lower prevalence of tungiasis, at 43%, the same as Tanzania and Cameroon, while the prevalence in studies conducted among primary schoolchildren in rural communities from Uganda and Rwanda was 23%.^16,17^ However, this prevalence is much higher than the rates reported in studies in Kenya. Community-based studies in Kilifi and western Kenya had prevalences of 21.5% and 25%, respectively, although in studies among school-age children the prevalence rates were slightly higher, ranging from 19 to 48%.^18,19^ However, the prevalence of tungiasis has been shown to be heterogenous from one community to another, even when the climatic, cultural, economic and social factors are similar.^20–22^ This, coupled with the different research methods adopted by various studies, accounts for the variances in prevalence.

Several risk factors have been associated with jiggers infestation. In this study, the fathers’ education level, residence (village) and wearing shoes were found to be significantly associated with tungiasis infection. No or low literacy as a risk factor has been elucidated in previous studies.^15,23^ Most of these studies used the mothers’ education level, unlike this study where the fathers’ education level was identified. Children whose parents are illiterate or with a low (primary) education level tend to have higher odds of tungiasis compared with those whose parents attended secondary or tertiary-level education. Education level determines the economic level of the family and the availability of resources such as shoes and better housing, which are associated with the prevention of tungiasis infection.^1^

Residence or the village in which the children reside affected the possibility of having tungiasis. Children residing in Dzombo B and Kiyungu had a lower prevalence of tungiasis compared with other villages. This factor was also identified by Wiese et al.,^19^ who found considerable variation in the prevalence and severity of tungiasis in villages due to demographic, socio-economic, environmental and geographic factors, although our study did not undertake similar extensive data analysis.

Other factors, although not significant but prevalent in this study, included the type of flooring used in the home and keeping animals, which was a common practice, especially in tungiasis-infected households. Earthen (sand) floors are quite common in Lunga Lunga subcounty, where most of the participants lived in houses with earthen/sand floors and kept livestock, although this was not found to be significant in this study. It is known that earthen floors provide a favourable environment for sand fleas. The fleas live 2–4 cm under the sand and thus household members are likely to get tungiasis.^16,17,21^

Animals act as reservoirs for sand fleas, promoting the transmission of tungiasis in humans. Although several studies have found animals to be involved in the transmission of tungiasis, studies done in coastal Kenya showed that tungiasis might not involve animal reservoirs in this region. This could be attributed to the types of animals that are kept in coastal Kenya. In West Africa and Brazil, communities keep pigs, cats, rodents and dogs, which have been found to be highly infected with *T. penetrans*, increasing the risk of transmission to humans. However, in coastal Kenya, the main animals kept are goats, poultry and cattle. The current study indicates low tungiasis prevalence, hence lower transmission levels.^4,24,25^

Wearing shoes is a critical measure for prevention of flea infestation. This study found that not wearing shoes was significantly associated with having tungiasis, as the odds of having tungiasis were 1.9 times higher among those not wearing shoes compared with those who wore shoes. Less than 30% of the school-age children wore footwear regularly. Promoting regular wearing of shoes is vital in the control and prevention of tungiasis. Children not wearing shoes are exposed to contaminated soil, predisposing them to flea penetration and infestation, especially in classrooms and playgrounds, where there is an increased potential for parasite carriers due to the human traffic involved.^26^

Consistently wearing shoes may help in preventing the onset or progression of a wide range of neglected tropical diseases (NTDs) and interest is growing regarding the use of footwear in the primary prevention of NTDs, including tungiasis.^27^

In this article, we understand that ‘adequate clothing’ includes footwear, and hence its absence results in an inadequate standard of living. This is contrary to article 25 of the Universal Declaration of Human Rights, which states that ‘everyone has the right to a standard of living adequate for the health and well-being of himself and of his family, including food, clothing, housing and medical care and necessary social services’.^28^ This parallels article 43 of the Kenyan Constitution, which states that ‘every person has the right to the highest attainable standard of health, which includes the right to health care services’.^29^

The overall knowledge level on tungiasis was poor. Only 1 (1%) respondent was aware that fleas were the main cause of tungiasis, although most of households (85%) associated poor hygiene and the presence of dust in the households or community as the cause or source of transmission of tungiasis. This community's understanding of tungiasis is better than in studies done in western and central Kenya, where the cause of tungiasis is often associated with witchcraft and curses.^18,30^ Knowledge of symptoms is a precursor for household members to seek treatment and implement preventive measures to control the spread of tungiasis infection. The respondents had a good knowledge of symptoms and prevention measures, as most of them identified the symptoms as itching, pain and blackspots, which are the most common symptoms of tungiasis.^4,31^ Good hygiene, wearing shoes, plastering floors and daily feet inspection are key preventive measures for tungiasis identified by the community. The control and prevention of tungiasis relies on a proper understanding on the cause and transmission of the disease, which translates into improved treatment prevention and control practices in the community and households. Therefore this information gap, coupled with the high prevalence, implies that tungiasis is a major problem in Kwale County and efforts are needed to undertake community sensitization and education regarding the causes, treatment, prevention and control of tungiasis.

**Figure 1.**
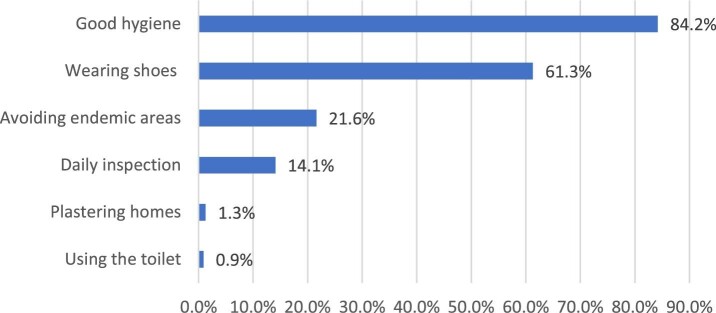
Tungiasis prevention.

**Figure 2.**
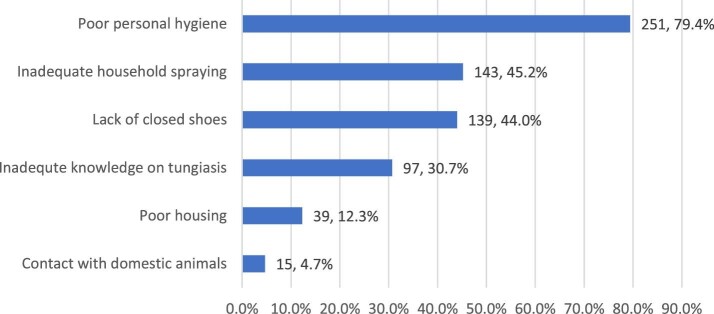
Reasons why tungiasis is a serious problem.

With regards to the impact of having tungiasis, the symptoms experienced included lack of sleep and poor concentration, resulting in absenteeism from school and failure to play or participate in daily chores. Due to the community perception that the condition is contagious or associated with poor hygiene, the affected children suffered from social stigma, altering the children's relationships and interactions with friends and peers. Children with tungiasis are often teased and ridiculed due to the shame associated with having tungiasis, especially since it affects mostly the poor and the skin alterations are visible. This is similar to observations made by Quaife et al.,^32^ where children are discriminated against due to tungiasis, affecting their social and academic lives.

For treatment and management practices following tungiasis infestation, extraction or removal was a common practice among the respondents, while other participants applied products with/without extraction. The standard recommended treatment for tungiasis is usually extraction in a sterile environment at a health facility or soaking feet in potassium permanganate for 10 min. As shown in previous studies, self-extraction or extraction by a caregiver is commonly practiced in Kenya and other countries such as Brazil and Uganda, although it is not recommended due to the associated risks of infection, haemorrhage and transmission of viral infections like hepatitis B and C and human immunodeficiency virus. Other than extraction, application of substances like coconut oil, palm oil, motor oil, topical products, medical ointments and insecticide is popular despite the possibility of toxic products being used.^33^ This indicates that households do not follow appropriate treatment practices, which can be attributed to a lack of knowledge, lack of access/costs associated with visiting health facilities, potassium permanganate not being effective as a treatment option or convenience of self-extraction/application of products at home.

Healthcare providers and community health workers should provide education on early identification of tungiasis symptoms and safe tungiasis treatment practices, provide the communities with potassium permanganate for use at home and encourage individuals to go to a health facility for surgical extraction.

## Conclusions

The present study examined the factors influencing prevention and control of tungiasis infection among school-age children in Kwale County, Kenya. Creating awareness of the cause of tungiasis remains of key public importance if prevention of this NTD is to be achieved. Hygiene promotion, including wearing shoes, and the general cleanliness of the environment at the community level using the community health structure should be implemented.

## Data Availability

The data used to support the findings of this study are included within the article.

## Notes

The authors would like to acknowledge the Director of the Kenya Medical Research Institute (KEMRI) for leadership during the research activity. Thanks goes to the study participants and the study team as a whole. We wish to express our sincere thanks to the county administrators in Kwale, staff of the Ministries of Health and the community members for facilitating the smooth running of the project in Lunga Lunga subcounty. We are also grateful to the young respondents for giving us an opportunity to talk to them. This article is published with the permission of the Director of KEMRI.
